# Chronic interfacing with the autonomic nervous system using carbon nanotube (CNT) yarn electrodes

**DOI:** 10.1038/s41598-017-10639-w

**Published:** 2017-09-15

**Authors:** Grant A. McCallum, Xiaohong Sui, Chen Qiu, Joseph Marmerstein, Yang Zheng, Thomas E. Eggers, Chuangang Hu, Liming Dai, Dominique M. Durand

**Affiliations:** 1 Neural Engineering Center, Department of Biomedical Engineering, Case Western Reserve University, Cleveland, OH 44106-7078 USA; 2 Department of Macromolecular Science and Engineering, Case Western Reserve University, Cleveland, OH 44106-7078 USA; 3 School of Biomedical Engineering, Shanghai Jiao Tong University, Shanghai, 200240 China

## Abstract

The ability to reliably and safely communicate chronically with small diameter (100–300 µm) autonomic nerves could have a significant impact in fundamental biomedical research and clinical applications. However, this ability has remained elusive with existing neural interface technologies. Here we show a new chronic nerve interface using highly flexible materials with axon-like dimensions. The interface was implemented with carbon nanotube (CNT) yarn electrodes to chronically record neural activity from two separate autonomic nerves: the glossopharyngeal and vagus nerves. The recorded neural signals maintain a high signal-to-noise ratio (>10 dB) in chronic implant models. We further demonstrate the ability to process the neural activity to detect hypoxic and gastric extension events from the glossopharyngeal and vagus nerves, respectively. These results establish a novel, chronic platform neural interfacing technique with the autonomic nervous system and demonstrate the possibility of regulating internal organ function, leading to new bioelectronic therapies and patient health monitoring.

## Introduction

The autonomic nervous system provides non-directed, unconscious control over bodily functions, such as digestion, breathing and heartbeat. Interfacing with this system to first detect a physiologic anomaly from the neural activity and then modulate its signaling behavior using electrical stimulation could create revolutionary diagnostic and therapeutic modalities targeting chronic health conditions and diseases^1–10^. However, these autonomic nerve bundles (fascicles) have small diameters (100–300 µm) and are contained within a mechanically strong and a highly, electrically insulated membrane called the perineurium^11^. In addition to the implant device biocompatibility, these limitations make it challenging to create a chronic, neural interface that is both reliable and safe for implantation. The leading theory why chronic neural interfaces fail is the mechanical mismatch between the implant and the surrounding biological tissue, causing micromotion between the two media^12^. This triggers the body’s natural inflammation process enhancing the fibrotic encapsulation barrier between the implant and the neural tissue and can result in recording signal losses with higher stimulation current thresholds that could damage the surrounding tissue^13^. Another important consideration is the dimension of the implant. Recent data in the central nervous system (CNS) indicate that decreasing the size of the implant generates little or no reaction^14^. Therefore, two important specifications are that small nerve implants should have axon-like dimensions and flexibility.

Cuff electrodes are the most widely used neural interface type based on their perceived safety compared to other neural interface implementations, ease of implantation and clinical results from nerve stimulation trials^15,16^. A true, feedback-based neuromodulation system must be able to successfully record and process neural activity with a high signal-to-noise ratio (SNR), which requires a stable, low electrical impedance between the recording electrode and the neural tissue. However, extrafascicular cuff electrodes fail to produce neural recordings that are highly selective with high SNR because they detect weak, local field potentials outside the electrically insulating perineurium layer^17^. Others have attempted to record chronically using wire hook electrodes around sympathetic nerves but the signals they acquire can be highly contaminated with triboelectric noise^18–20^ (Supplementary Fig. 5). To address these limitations, microfabrication techniques have been employed to create intrafascicular electrodes designed to penetrate the perineurium and place the electrode contacts in closer proximity to the nerve fibers. The three most prevalent designs are: (1) the longitudinal intrafascicular electrode (LIFE)^21^, which is sewn into the nerve along its length; (2) the transverse intrafascicular multi-channel electrode (TIME)^22^, which penetrates through the nerve’s cross-section; and (3) the Utah Slanted Electrode Array (USEA)^23^, which is an array of multi-height, cone-shaped, needle structures that penetrate the nerve similar to the TIME. Although these intrafascicular electrodes have been able to initially record high SNR neural signals^24,25^, they have yet to demonstrate chronic implant reliability because, we hypothesize, the electrode flexural rigidity is at least five orders of magnitude greater than the surrounding neural tissue^14,26^. To achieve chronic neural recordings with high selectivity and SNR, a neural interface should satisfy three important design criteria: high flexibility, small dimensions and intrafascicular penetration. To achieve such a design, we used carbon nanotube fabrication techniques to create an intrafascicular CNT yarn (CNTY) interface. The CNT yarn possesses an electrical conductivity and flexural rigidity that is superior to existing intrafascicular implementations. To insert such a flexible material such as a CNT yarn into a nerve, we have developed a novel implantation method based on microneurography techniques. We have chronically implanted this new electrode into the rat vagus and glossopharyngeal nerves to determine if gastric extension and hypoxic events can be identified based on the chronically recorded autonomic nerve activity. Preliminary chronic stimulation experiments were performed on the rat sciatic nerve and charge thresholds were recorded over a six week period.

## Results

### Chronic recordings of autonomic nervous system activity

**Figure 1:**
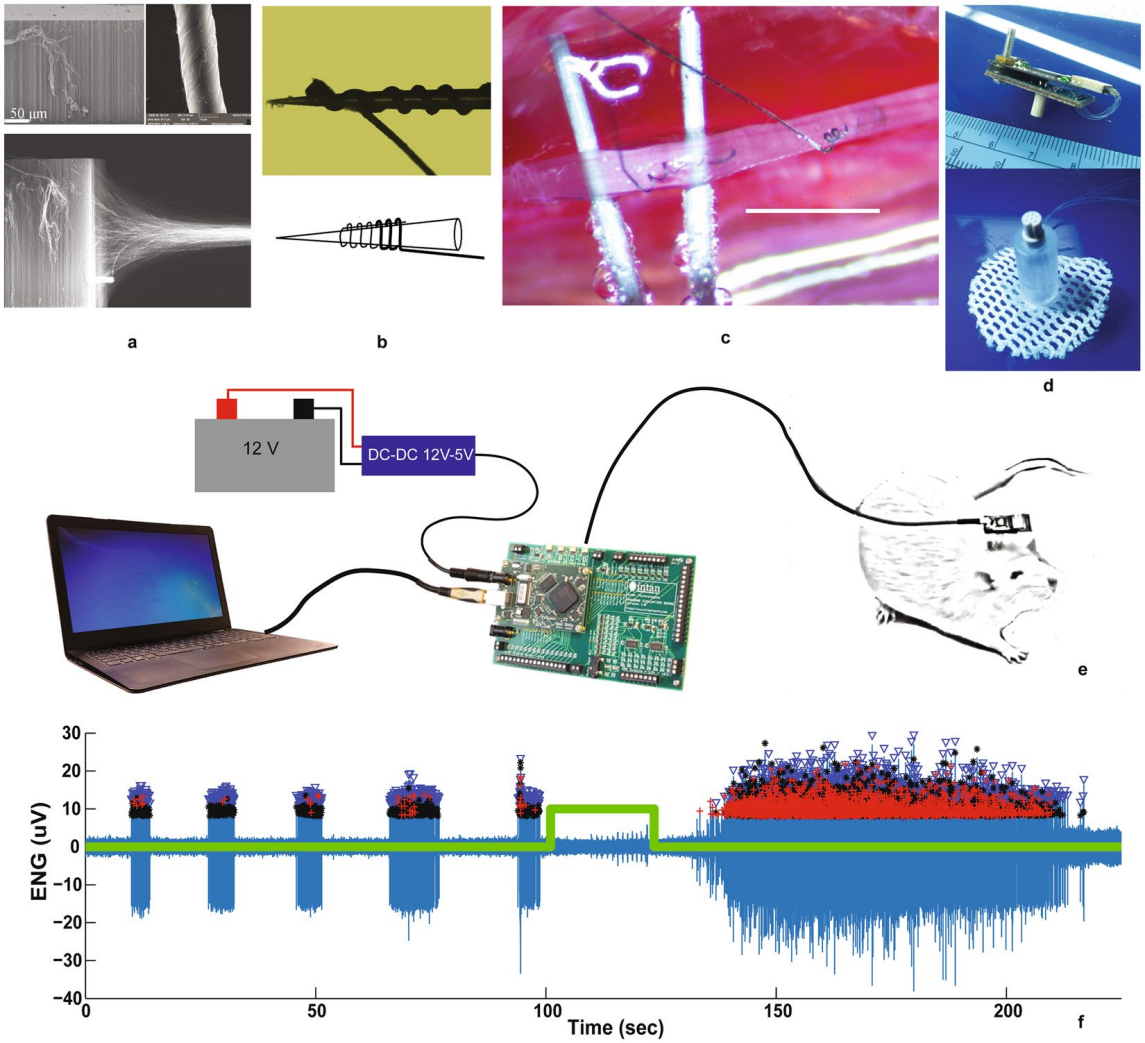
Chronic autonomic nerve recording using CNT yarns. (**a**) CNT forest being pulled and spun to form CNT yarns that are 10 microns in diameter. (**b**) CNT yarns wound around the tip of a Tungsten microneedle used to implant the yarn into the nerve. (**c**) Two CNT yarns implanted in the rat vagus nerve separated by ~2 mm for differential recording of the neural activity [sc﻿ale bar = 2 mm]. (**d**) Percutaneous plug with electrical connector that directly mates to the neural amplifier recording circuit board for signal amplification, filtering and acquisition. (**e**) Schematic of the complete recording assembly from the CNT yarn implants, lead wire connections, percutaneous plug, the external neural recording amplifier board and evaluation system board for data storage. (**f**) Post processed, spontaneous neural activity from the vagus nerve, 16-weeks post implant (blue). Trigger signal (green trace) indicating the hypoxia event administered to the anesthetized rat. Also shown are the markers for each identified neural spike and cluster grouping for activity identification and classification. Evaluation system board photo used with permission courtesy of Intan Technologies, LLC.

**Figure 2:**
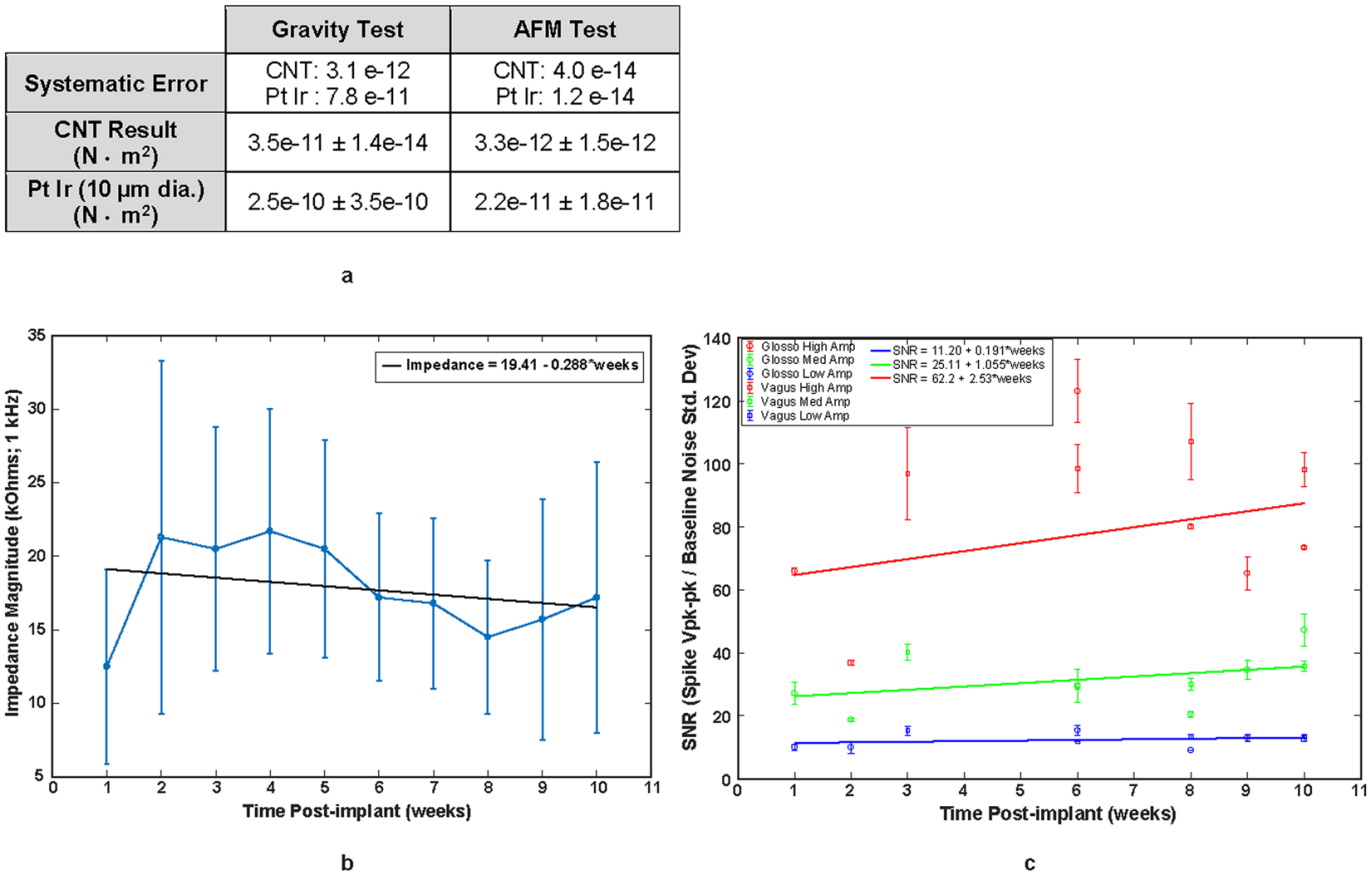
Flexural rigidity, chronic impedance and SNR measurements. (**a**) Flexural rigidity measurements comparing CNTY to platinum-iridium (PtIr) wire of the same diameter (10 micron). Two measurement methods were used, gravity and atomic force microscopy (AFM) and in both cases the CNTY had a flexural rigidity that was an order of magnitude less than the PtIr wire. (**b**) Impedance magnitudes measured at 1 kHz over a 10 week implant period. (n = 10 total CNTYs implanted in five separate subjects). Regression analysis shows that the slope of the impedance line is not significantly different from zero indicating a stable implant impedance over the 10 week period. (P-value = 0.428) (**c**) SNR measured over 10 week implant period in three different subjects and separated into three groups: High, Medium and Low spike amplitudes. Regression analysis also shows a stable SNR value for the High, Medium and Low spike amplitude groups as their regression line slopes are not significantly different from zero. (P-values = 0.420, 0.298 and 0.448, respectively).

Achieving chronic recordings from small diameter autonomic nerves requires both novel electrode development and implantation process (Fig. 1a–c). Owing to their excellent electrical, thermal, and mechanical properties, carbon nanotubes have attracted a great deal of interest for various applications, particularly as new flexible electrical wires in advanced biomedical systems. However, the connection of nanotubes in biomedical systems to the outside world is challenging. Recent efforts have led to the creation of CNT yarns by continuous, high-rate spinning vertically-aligned multi-walled CNT (VA-MWCNT) arrays^27^ (Fig. 1a). The availability of flexible, mechanically strong, and highly conducting CNT yarns with micro-sized diameters has offered ideal alternatives to replace rigid and expensive noble metal wires. Compared to platinum iridium wires of identical diameter, the CNT yarns are both 10 times more conductive and more flexible (Fig. 2a and﻿ Supplementary Fig. 1a). These two parameters are of critical importance for interfacing with small diameter nerves since the high conductance will reduce the electrical baseline noise in the neural recordings and the flexibility will be more mechanically similar to the surrounding tissue, reducing nerve damage and the inflammation response.

The CNT yarns were coated with a thin insulating layer except for a ~500 µm long segment at one end. This uninsulated segment is wound around the tip of a Tungsten microneedle similar to those used in clinical microneurography procedures^28^ (Fig. 1b). Under sterile surgical conditions, a portion of the target nerve was dissected away from all connective tissue. The microneedle loaded with the CNT yarn electrode was then advanced into the nerve until the exposed yarn segment was completely inside. The microneedle was then pulled back out of the nerve leaving the CNT yarn electrode embedded within (Fig. 1c, Supplementary Fig. 3b). For each target nerve, two CNT yarn electrodes were implanted within a 2 mm separation distance. Fibrin glue was then applied to completely surround the nerve at the implant sites to help secure the CNT yarns in place. The opposite ends of the electrode directly interfaced to a percutaneous connector. A removable neural amplifier recording board was used to amplify, filter, digitize and store the neural activity to a laptop computer (Fig. 1d, Supplementary Fig. 4c). Figure 1e depicts the entire neural recording assembly from the implanted CNT yarns to the external data acquisition computer. Spontaneous, multi-unit neural spike activity recorded using CNT yarn electrodes after being implanted in the vagus nerve for four months (Fig. 1f) shows signal amplitudes ranging from 30–60 µV peak-to-peak.

Furthermore, signal processing techniques can be applied to these recorded neural waveforms to identify individual spike activity based on a desired threshold level, clustering algorithms to separate the activity into groups based on the waveform shape and temporal firing patterns, and ultimately determine the firing rate for each identified neural group (Fig. 1f).

Figure 2 summarizes three important points regarding this interface technology: (1) the flexural rigidity value of the CNT yarn electrode compared to a Platinum-Iridium (PtIr) wire of a similar 10 µm diameter is an order of magnitude lower; (2) the measured, *in vivo*, chronic impedance values are stable over time; (3) the resulting SNR from the recorded waveforms is also stable over time. As shown in Fig. 2a, the flexural rigidity was measured using two methods, gravity and atomic force microscopy (AFM). In both cases, the flexural rigidity of the CNT yarn was found to be an order of magnitude less when compared to the PtIr wire demonstrating its superior flexibility compared to traditional electrode materials^29^. Figure 2b shows the CNTY 1 kHz impedance magnitude measurements over a 10 week, post implant period. Data was collected from five different subjects each with two implanted CNT yarns (n = 10). The average impedance value over the 10 weeks was 17.8 ± 7.7 k-Ohms (average ± std. dev). Linear regression was performed on the data and a t-test was run on the regression line slope. The results determined the slope was not significantly different from zero (p = 0.428) indicating a stable impedance magnitude over the 10 week period. Similarly, the SNR over a 10 week period is shown in Fig. 2c from three different subjects. The recorded spikes were analyzed separately in three different amplitude groups: High, Medium and Low. For each group, the SNR was calculated by taking the spike peak-to-peak voltage levels and dividing it by the baseline standard deviation value in a period with no neural activity. For the three SNR groups high, medium and low, the average SNR was found to be 62.2 ± 2.5, 25.11 ± 1.1 and 11.20 ± 0.2 (average ± std. dev.), respectively. Linear regression was also performed to determine if each SNR group’s regression line slope was significantly different from zero. The results indicate none were significantly different from zero with P-values of 0.420, 0.298 and 0.448 for the high, medium and low SNR groups, respectively.

### Chronic recordings of glossopharyngeal nerve activity

**Figure 3:**
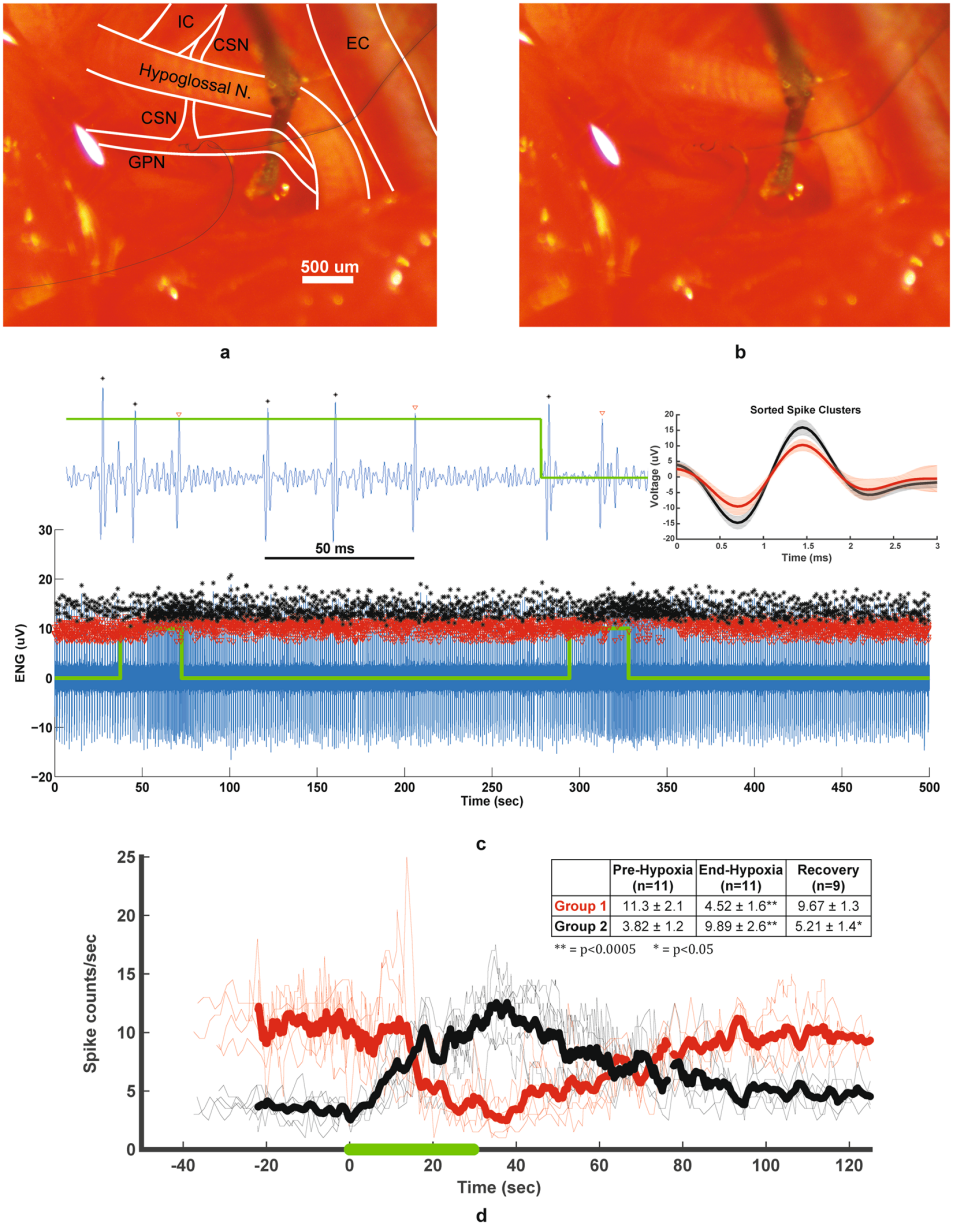
Chronically recording activity from the glossopharyngeal nerve. (**a**) Diagram of the two CNT yarn implants in the glossopharyngeal nerve (GPN) near the junction point with the carotid sinus nerve (CSN) that runs beneath the hypoglossal nerve. Also, shown is the external and internal carotid arteries (EC and IC), respectively. (**b**) Original picture of CNT yarn implants in the GPN. (**c**) 16 day post-implant electroneurogram (ENG) recordings obtained from the CNT yarn electrodes during two hypoxia events (green trace). Post-processing identifies two distinct neural groups (red and black markers). Magnified plot showing the neural spike activity at the end of the second hypoxia event. (**d**) Rate of neural spike activity showing the baseline activity and the repeatable change in activity for each group during six of eleven total hypoxia events (thin traces), as well as the averaged spike activity (bold lines). Green line represents hypoxic challenge. Table insert shows the firing rates (averaged over 5 seconds) for each group before hypoxia, end of hypoxia, and after a 90 second recovery period. End and recovery rates were compared to pre-hypoxia firing rates, with **p < 0.0005, and *p < 0.05.

The glossopharyngeal nerve (GPN) is known to contain afferent and efferent nerve fibers that innervate the upper airways, taste and temperature receptors on the tongue, and chemoreceptor afferents that originate in the carotid body and baroreceptor afferents that emanate from the carotid sinus and join the GPN through the carotid sinus nerve (CSN) (Fig. 3a)^30^. Chemoafferent activity from the carotid body and its role in cardiovascular and respiratory diseases has received considerable interest^31–33^. Increased activity of carotid body chemoreceptors have been linked to sleep apnea and heart failure^34–36^. In addition, chemoreflex-evoked sympathetic responses are enhanced in humans and animal models of systemic hypertension^37–40^. We have investigated the GPN’s response to a hypoxic challenge given to an anesthetized rat that has been implanted with CNT yarn electrodes for 16 days (Fig. 3b). The recorded neural activity is presented with an overlay of the hypoxic events in which the gas used to anesthetize the rat was switched to a highly concentrated nitrogen gas (90%/10%, nitrogen/oxygen) until the animal’s blood oxygen saturation percentage was reduced to 75%. The gas was then switched back to the anesthetic gas, allowing the rat to recover. In some cases, a second hypoxic event was administered following recovery (Fig. 3c). A total of 11 hypoxic events were administered at 7, 10, and 16 days post implant.

The post-processed data revealed two distinct groups of neural activity (Fig. 3c) with the individual spike signals shown in the magnified trace and again in the sorted spike clusters plot for a single experiment, shaded regions indicated the standard deviation for each cluster. Each group’s firing rate over the course of six experiments is displayed in Fig. 3d, aligned with the onset of hypoxia at time = 0. During several experiments, it took an extra 10 seconds for the rat to reach 75% blood oxygen saturation; thus the data from these experiments is excluded from Fig. 3d, though it is still used in the statistical analyses of firing rates. The table in Fig. 3d shows the firing rates for each of the two spike groups (±standard deviation) immediately before the onset of hypoxia, at the end of the hypoxic event, and approximately 90 seconds after the hypoxic event. Experiments 1 and 2 did not have sufficient recovery data recorded, but were still included in the analysis for the firing rates before and at the end of hypoxia.

**Figure 4:**
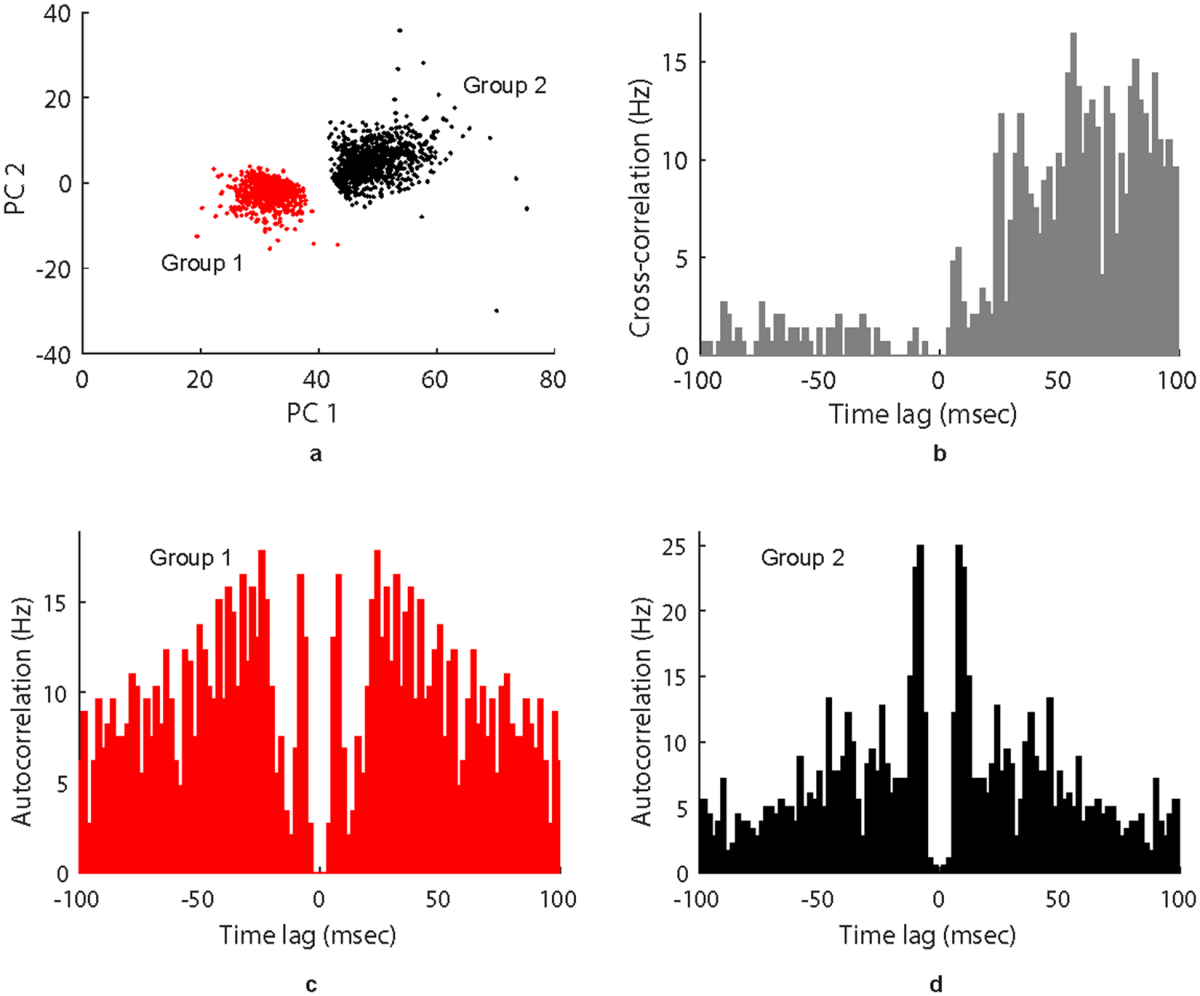
Spike cluster separation 10 days post-implant. Several spike separation and quality metrics calculated from data collected 10 days after electrode implantation. (**a**) Representation of clusters 1 and 2 in the feature space, showing only the first two principal components for each spike. (**b**) Cross-correlation of spike times between clusters 1 and 2; bin width is 2 ms. (**c**) Auto-correlation of spike times from cluster 1; bin width is 2 ms. (**d**) Auto-correlation of spike times from cluster 2; bin width is 2 ms.

Cluster separation was analyzed using the UltraMegaSort2000 MATLAB software package^41^. Quality metrics (false positive and false negative events) for both clusters are shown at 7, 10, and 16 days in Tables 1 and 2. Figure 4a shows separation of the two groups in the first two principal components at 10 days post-implant; Supplementary Figs 6a and 7a show the feature-space of the two groups at 7 and 16 days, respectively. Figure 4b shows cross-correlation of the spike times of the two clusters at 10 days, with Supplementary Figs 6b and 7b showing cross-correlation of spike times at 7 and 16 Days. Finally, Fig. 4c,d, and Supplementary Figs 6c,d, 7c and d show the autocorrelation of each cluster’s spike times at 7, 10, and 16 days.

**Table 1:** Summary of cluster statistics for units in Fig. 3: false-positive events.

Day		Refractory violations, ${{\boldsymbol{f}}}_{{\bf{1}}}^{{\boldsymbol{p}}}$	Overlap of clusters, ${{\boldsymbol{f}}}_{{\bf{2}}}^{{\boldsymbol{p}}}$	Composite, max (${{\boldsymbol{f}}}_{{\bf{1}}}^{{\boldsymbol{p}}},{{\boldsymbol{f}}}_{{\bf{2}}}^{{\boldsymbol{p}}}$)
7	Group 1	0.1143	0.0002	0.1143
	Group 2	0	0.0022	0.0022
10	Group 1	0	0.0029	0.0029
	Group 2	0.1646	3.24e-06	0.1646
16	Group 1	0	0.0343	0.0343
	Group 2	0	0.0328	0.0328

**Table 2:** Summary of cluster statistics for units in Fig. 3: false-negative events.

Day		Undetected spikes, ${{\boldsymbol{f}}}_{{\bf{1}}}^{{\boldsymbol{n}}}$	Overlap of clusters, ${{\boldsymbol{f}}}_{{\bf{2}}}^{{\boldsymbol{n}}}$	Censored spikes, ${{\boldsymbol{f}}}_{{\bf{3}}}^{{\boldsymbol{n}}}$	Composite: [1−(1 − ${{\boldsymbol{f}}}_{{\bf{1}}}^{{\boldsymbol{n}}}$) (1 − ${{\boldsymbol{f}}}_{{\bf{3}}}^{{\boldsymbol{n}}}$)] + ${{\boldsymbol{f}}}_{{\bf{2}}}^{{\boldsymbol{n}}}$
7	Group 1	0.1208	0.0022	0.0058	0.1280
	Group 2	0	0.0004	0.0066	0.0070
10	Group 1	0.1713	4.01e-06	0.0041	0.1750
	Group 2	7.15e-15	0.0024	0.0039	0.0064
16	Group 1	0.1472	0.0288	0.0033	0.1788
	Group 2	0.0021	0.0391	0.0035	0.0447

The Group 1 firing rate decreased significantly from before the onset of hypoxia to the end of the hypoxic event (p = 0.00036), and that firing rate increased significantly from the end of the event to end of the 90 second recovery time (p = 0.0023). However, the firing rate was not significantly different between the pre-hypoxia and post-recovery measurements (p = 0.34). Similarly, the Group 2 firing rate increased significantly from before the onset of hypoxia to the end of the hypoxic event (p = 0.00019), and that firing rate decreased significantly from the end of the event to end of the 90 second recovery time (p = 0.0018). Additionally, the firing rate at the end of the recovery period was slightly higher than the firing rate before the hypoxia was administered (p = 0.012).

We hypothesize that Group 1 could be baroreceptor activity as the blood pressure is known to decrease for anesthetized rats under hypoxia^42^, which reduces baroreceptor activity. Furthermore, Group2 could be chemoreceptor activity based on their known behavior of increased activity during falling blood oxygen level concentrations^43^. This experiment was performed on three different days over a 16 day period with identical results, demonstrating the possibility of identifying hypoxic events from recorded neural activity in the glossopharyngeal nerve.

### Chronic recordings of vagus nerve activity

The vagus nerve (VN) contains fibers that innervate many internal organs, including the stomach and digestive system^44^. It is likely that vagal activity plays an important role in a variety of pathologies, such as irritable bowel syndrome (IBS). We investigated vagal response to a stomach distention^45–47^ given to an anaesthetized rat that has been implanted with CNT yarn electrodes in the left cervical vagus for up to 50 days. The rat was fasted overnight before the procedure, and then a tube was inserted into the rat’s stomach. 3 mL of sterile saline was infused into the stomach and the tube was immediately removed to prevent inhalation of saline into the lungs. Vagal activity was recorded for several minutes before, during, and after the procedure.

**Figure 5:**
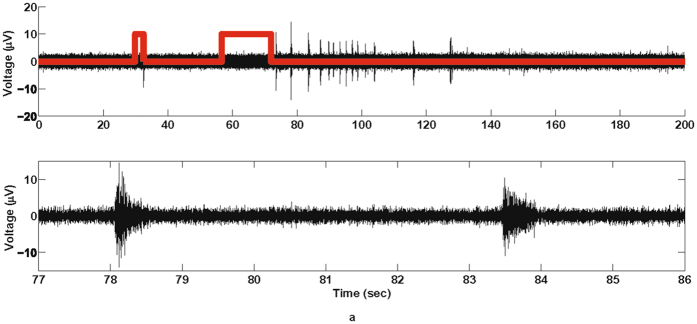
Chronically recording activity from the cervical vagus nerve. (**a**) (Top) 48-day post-implant electroneurogram recordings obtained from the CNT yarn electrodes during a gastric extension experiment (red trace). Short pulse indicates when tube primed with 1 mL of saline and longer pulse indicates when 3 mL of saline introduced into the stomach. (Bottom) Expanded view of the neural activity during the gastric extension.

In response to the gastric distention, we observed intermittent bursting behavior (Fig. 5) during and after saline infusion into the stomach. These bursts appeared to occur randomly in response to the gastric stimuli, but were observed each of the five times the experiment was conducted over a 21-day span (40 to 61 days post-implant). The repeatability of this result illustrates the possibility of identifying patient risk for pathological gastrointestinal responses via vagal activity, or patient responses to treatments and therapies. In addition, due to the nearly ubiquitous innervations of the VN, this technology may be useful in identifying responses to other stimuli, including hypoxia, changes in heart rate or blood pressure, or bronchoconstriction. It may also be possible to specifically target specific branches of the VN to isolate physiological responses.

### Chronic CNT nerve histology using CLARITY

**Figure 6:**
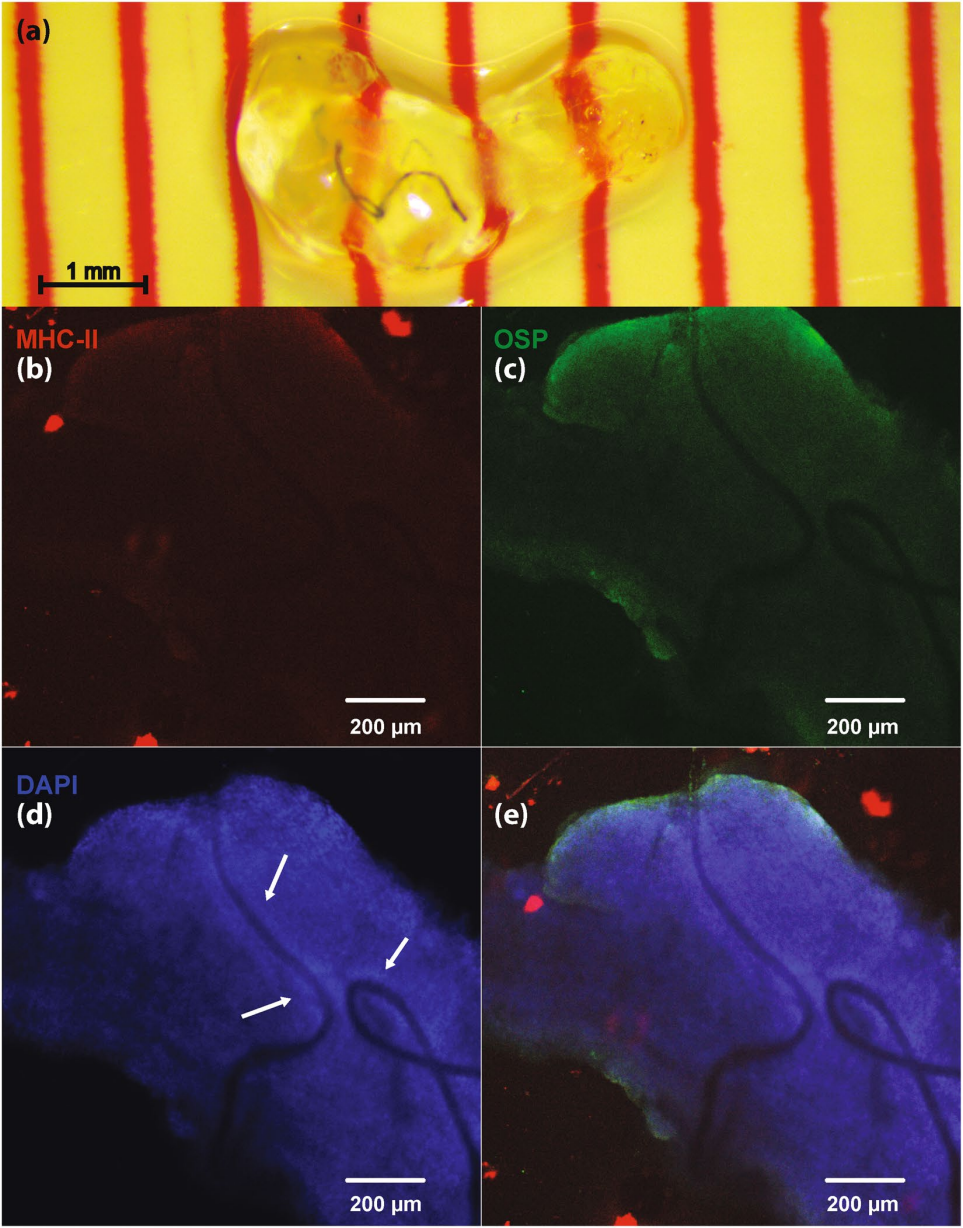
Immunohistology for a chronically implanted VN. Fluorescent images shown at 10x magnification. (**a**) Cleared VN with implanted CNT yarn electrodes. Nerve shown after 21 days of passive clearing. (**b**) Fluorescent stain for major histocompatibility complex class II (MHC-II, ab55152), present on antigen-presenting immune cells (e.g. activated microglia). (**c**) Fluorescent stain for oligodendrocyte specific protein (OSP, ab7474). (**d**) Fluorescent stain for DNA (DAPI). DAPI is used to stain cell nucleii. Arrows show elevated DAPI density around the wires, used to estimate thickness of fibrous encapsulation. (**e**) Composite image showing MHC-II, OSP, and DAPI stains.

**Figure 7:**
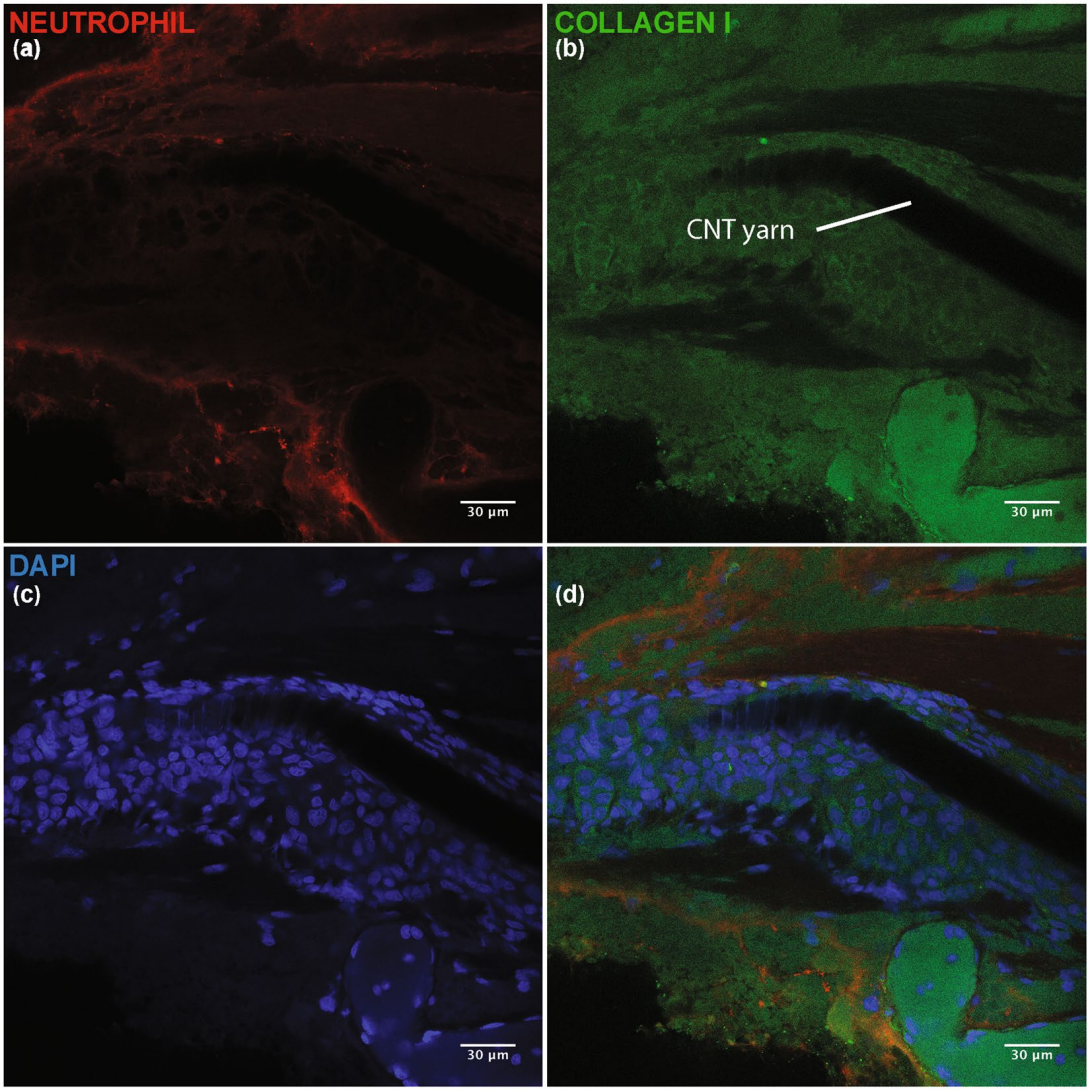
Immunohistology for chronically implanted GPN. Images shown at 40x magnification. (**a**) Fluorescent stain for neutrophils (NIMP-R14, ab2557). (**b**) Fluorescent stain for Collagen I (ab24133). Collagen makes up a significant portion of fibrous encapsulation. (**c**) Fluorescent stain for DNA (DAPI). DAPI is used to stain cell nucleii. (**d**) Composite image showing neutrophil, collagen I, and DAPI stains.

Figure 6 shows a VN implanted with a CNT yarn electrodes for seven days. Figure 7 shows a GPN implanted with CNT yarn electrodes for 138 days. Figure 6b shows no evidence of response from antigen-presenting immune cells (activated microglia) after one week. There is no significant localized expression of oligodendrocyte specific protein (OSP, shown in Fig. 6c), suggesting a small population of Schwann cells, and thus no significant nerve injury. Similarly, Fig. 7a shows a lack of localized neutrophil activity, which suggests a lack of prolonged inflammatory response after chronic implantation. DAPI and collagen I show a thin layer of cellular encapsulation around the CNT yarns (Figs 6d, 7b, and c); thickness was estimated to be 35.0 ± 2.67 µm in the VN, and 31.7 ± 4.5 µm in the GPN.

To estimate the thickness of the layer of encapsulation surrounding the implant, the perpendicular distance from the Parylene-C structure to the rim of the capsule (visually identified by change in DAPI/Collagen I density) was measured at several points along the electrode implant. Several measured locations are shown in Fig. 6d.

### Chronic CNT yarn electrode stimulation charge thresholds

**Figure 8:**
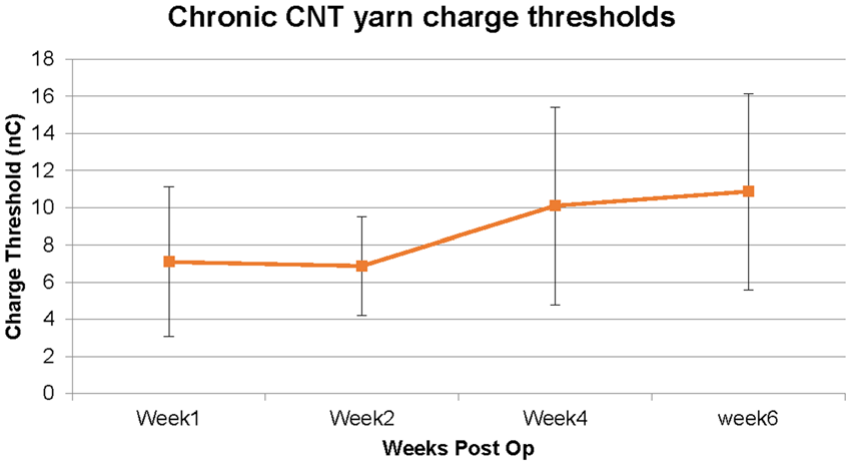
Chronic charge threshold values of CNT yarn electrodes. Charge threshold stimulation values measured in the peroneal and tibial nerve branches of five rats over a six week duration. Charge-balanced biphasic rectangular pulses of 100 microseconds were applied at 1 Hz with varying pulse amplitudes until contraction was seen; thresholds were converted to charge (nC) and plotted over time. The first four weeks contains five animals (ten thresholds, one for each fascicle), while week 6 contains 2 animals.

To test the ability of the nanowire interface to stimulate nerves, CNT wires were implanted in the tibial and peroneal branches of the sciatic nerve in five rats. Isolated current stimulation pulses of charge balanced, 100 µsec pulse width per phase were applied with variable amplitude to activate the nerve. The threshold for activation was estimated by detecting muscle twitches in the medial gastrocnemius and tibialis anterior muscles. Figure 8 shows the charge threshold measured from the sciatic nerve branches for five rats through 4 weeks and 2 rats at six weeks. The results across animals were fairly consistent, with similar thresholds from week 1–2 followed by an increase in threshold by week 4. Much of this change could be attributed to a single wire pair (out of 10) with a threshold increasing from 4 to 25 nC. It is unclear if this represents an increase due to natural encapsulation of the electrode, or if this electrode was somehow displaced within the fascicle. Regression analysis revealed that the trend line had a small but statistically significant positive slope over the six week period (p < 0.05).

## Discussion

We have developed the first successful use of CNT yarns to be implanted intrafascicularly to record/stimulate autonomic nerve activity in long-term chronic rats. The CNT yarns are fabricated using a dry-spinning method^27^, which is superior to chemical bath, wet-spinning methods^48^ for this specific application as the latter could leave behind non-biocompatible residues detrimental for long term implantation. The CNT yarns were connected to a more mechanically robust DFT wire to enable tunneling to a percutaneous port. The entire electrode implant assembly was insulated with Parylene-C using vapor deposition except for a small portion of the distal end for nerve implantation and the proximal end to connect to an external electrical connector. The CNT yarns were chronically implanted into two different autonomic nerves and electroneurogram data was collected during physiological challenges on rats, under anesthetized conditions, for up to 16 weeks. Statistical analysis of the measured impedance and SNR of the chronically implanted subjects, indicate that both metrics are stable over a 10 week implant period as shown in Fig. 2.

Chronic neural spike activity was successfully recorded from two separate autonomic nerves: glossopharyngeal and vagus. In both nerves the signal-to-noise ratio was over 10 dB for the duration of the implant. Different unit activity could be identified from the recordings and separated into distinct functional groups using classification and clustering algorithms off-line. This is clearly apparent in the glossopharyngeal recordings where two functional neural groups were repeatedly identified during hypoxia challenges throughout the duration of the study. The two spike groups appear to be distinct across 3 days of recording (7, 10, and 16 days post implant), with the first two principal components appearing relatively stable across those days (Fig. 4a, Supplementary Figs 6a, 7a). The cross-correlation of spike times for the two groups shows an upward trend (Fig. 4b); usually independent spike clusters would show a flat cross-correlation. However, the regular nature of the spiking activity (lined up with the respiratory rhythm), and its response to hypoxia, could account for the upward trend in the cross-correlation plot. The autocorrelation of spike times for each group (Fig. 4c,d) shows a small number of refractory violations for Group 1 and a slightly larger number of refractory violations for Group 2. These values vary day-to-day, but the fraction of refractory violations is less than 0.17 for both groups on all three days (Table 1). Due to the size of our electrodes, each of the spike clusters could contain spikes from more than one neuron, which could account for the refractory violations. We can see in Table 1 that the number of false positive events due to cluster overlap for at 7, 10, and 16 days remains small. In Table 2, we can see that Group 1 has a larger fraction of undetected spikes, which is expected since that group has smaller amplitude, which is close to our threshold. However, both clusters have a small fraction of censored and overlapping false negative events. The data shown in Fig. 4, Supplementary Figs 6, 7, and Tables 1 and 2 show that the two groups shown in Fig. 3 are distinct and repeatable over 3 recording days, up to 16 days post-implant. These two groups are believed to be chemoreceptor and baroreceptor activity based on the location of the CNT yarn implants where the carotid sinus nerve joins the glossopharyngeal nerve (Fig. 3). More investigation is needed to prove this claim is true, however, a hypoxic event can clearly and repeatedly be identified from the recorded neural spike activity. The vagus nerve activity proved to be more challenging to correlate to physiological function as the cervical vagus contains afferent fibers from a multitude of organ systems within the body. Each vagus implant yielded different neural activity patterns such as the spontaneous firings shown in Fig. 1, which are believed to be intestinal signals, or the elicited gastric extension activity (Fig. 5). Based on the implant depth, CNT yarn electrodes could detect activity within the entire cross-section of the nerve. Therefore, rather than implanting in the nerve trunk, targeting specific nerve branches that contain a limited number of functional nerve fiber groups would be ideal to identify specific physiological markers.

This new chronic electrode interface has significant benefits over the existing intrafascicular implants in regards to long-term stability. While some intrafascicular solutions have focused on using mechanically soft materials^21,22,49^ and others on implants with small cross-sectional areas but mechanically hard materials^23^, we hypothesize that to minimize the encapsulation process required for stable long-term intrafascicular interfaces one must design for a flexural rigidity as close as possible to that of the surrounding neural tissue. Any relative motion to the nerve caused by a non-flexible implant is believed to exacerbate the encapsulation process. Implanted CNT yarns have a lower flexural rigidity value (Fig. 2a) compared to existing intrafascicular electrodes as they have both a smaller second moment of inertia and lower modulus. Preliminary evidence of this is presented in Figs 6 and 7 as the cellular encapsulation observed in these CNT yarn experiments (~32 µm, 19 weeks post-implant) is smaller than the 135 ± 56 µm shown in the TIME electrodes^22^, the 113 ± 2.8 µm estimated for the thin-film LIFE, and the 64.3 ± 2.5 µm estimated for the PtIr LIFE^49^.

The results in Fig. 8 demonstrate the viability of using CNT yarn electrodes for chronic stimulation. While difficult to directly compare to other studies as authors tend to use varying pulse widths and stimulation frequencies, the injected charges here are slightly higher than previously published work investigating similar *in vivo* intrafascicular recording/stimulation but utilizing different electrodes^50^. The increase in threshold at week four is not unexpected as it typically marks the completion of the encapsulation layer formation and foreign body response to the implant. These thresholds stabilized at one month time point, although the data shown is limited to only six weeks post-op. However, three additional rats were implanted in the tibial nerve and left *in vivo* for 16 months. A single stimulation experiment was run on these animals, as their percutaneous connectors were destroyed early in the study. Of the four CNT yarn electrodes tested, three had normal impedance (~20 k) while two elicited twitches at relatively low thresholds, roughly 20 nC, versus ~70 nC for the other two electrodes. Together these results indicate that CNT yarn electrodes are relatively stable within the nerve and can be used for chronic stimulation. The charge capacity for stimulation is significant higher (~300 times) than that of platinum iridium wires^51^. Fundamentally, stimulating autonomic and somatic fibers is the same. The only difference would be differences in charge thresholds, with the unmyelinated fibers to likely require higher thresholds than reported for the somatic nerves. The data presented serves to demonstrate empirically that the CNT yarns can provide the necessary charge to activate nerve fibers, as well as to provide an additional metric for the stability of the interface over time.

The preliminary histology data and high quality neural recording results we have presented indicate that it is indeed possible to obtain information from the autonomic nervous system for long periods of time. This capability opens the possibility to reliably obtain and decode autonomic nerve signals to understand the physiological signaling mechanisms of the nervous system. The recording capability and the low stimulation charge thresholds indicate the feasibility of chronic implanted CNT yarn electrodes for autonomic nerve control and modulation to provide therapeutic and patient monitoring capabilities for various diseases and health conditions.

## Methods

### Carbon nanotube (CNT) yarn fabrication

VA-MWCNT arrays (with MWCNTs of *ca*.250 μm in length and *ca*.30 nm in diameter) (Fig. 1a), were first synthesized on Fe/Al-coated Si/SiO_2_ wafers using a low pressure chemical vapor deposition (CVD) method according to our previously reported procedure^52^. In a typical experiment, a 10-nm thick Al layer was coated on the silicon wafers before the deposition of 3-nm thick Fe film. The catalyst coated substrate was then inserted into a quartz tube furnace for the VA-MWCNT array growth using high-purity (99.99%) acetylene as the carbon source and Ar/H_2_ (2.5:1 v/v) as a carrier gas under 0.2 atm pressure at 760 °C^52^. For spinning VA-MWCNT arrays into the CNT yarn (Fig. 1a), one piece of the VA-MWCNT array of approximately 3-mm width on Si/SiO_2_ wafer was initially peeled off from the edge of the sidewall, drawn away from the CNT array, and attached onto a tip of the rod, which was connected to a motor^27^. A CNT yarn was spun by the rotating rod that was moved away from the array, and at the same time the bundle was rotated anti-clock-wise to form the CNT yarn (Supplementary Fig. 1b). The rotating speed was in the range of 2000–20000 rpm and the diameter of the as-prepared yarn was in the range of 10–20 μm, which was controlled by the number of CNTs initially pulled out from the array. The yarn diameter was determined by SEM images.

### CNT yarn electrode assembly process

The implant electrode assembly is comprised of two different wire types: CNT yarn for implantation in the nerve and a more robust lead wire used to route the electrode under the skin to a percutaneous electrical connector. The electrode lead wire is a 25 cm long (1 × 7 × 25.4 µm: 127 µm diameter) metal-to-metal composite wire with a silver core (28%) and a stainless steel outer tubing coated with PFA insulation (35NLT®-DFT® wire, Fort Wayne Metals). The DFT® wire insulation was stripped by approximately 3 mm at one end and joined to the CNT yarn by conductive epoxy resin (H20E, EPO-TEK). These wire assemblies were then placed and fixed on a custom-made acrylic rack (Supplementary Fig. 2a) for insulating the CNT yarn wire and junction. Parylene-C is chosen to be the insulation material for the CNT yarn. The Parylene-C thickness was 3.5 µm and was deposited using chemical vapor deposition (CVD). During the CVD coating process, a 500 µm portion of the CNT yarn end was left uninsulated by using tape to mask the desired area. After the Parylene-C insulation was deposited, the individual electrode wire assemblies were removed from the rack. The junction between the CNT yarn and the DFT® lead wire was placed within a 6 mm long (0.51 mm ID, 0.94 mm OD) silicone tube (2415500 - Dow Corning Corporation). Any empty space inside the silicone tube was filled with silicone elastomer (MED-4211/MED-4011, NuSil Silicone Technology) to further insulate the wire-to-wire junction.

Next, the uninsulated CNT yarn end and a short portion of the insulated yarn was wound around the tip of a Tungsten microneedle (UEWSGKSNXNND, FHC Inc.) (Fig. 1b). Then the silicone tube between the CNT yarn and DFT® wire was fixed to the microneedle using a slip knot made of thread (Supplementary Fig. 2b). After winding, 3 mm of DFT® wire insulation at the free end was stripped to be eventually soldered to a percutaneous electrical connector. A ground electrode (Supplementary Fig. 2c) was made with a 3 × 4 cm, 25 µm diameter Platinum-Iridium (Pt-Ir) wire and DFT® wire. The Pt-Ir wire and DFT® wire were joined together by conductive epoxy. This ground electrode was placed chronically under the dorsal skin of the animal.

### Chronic *In vivo* implantation procedure

All surgical and experimental procedures were done with the approval and oversight of the Case Western Reserve University, Institutional Animal Care and Use Committee to ensure compliance with all federal, state and local animal welfare laws and regulations.

Adult male Sprague Dawley rats (350–450 grams, Charles River Laboratories) were used for all peripheral nerve implants discussed. Prior to surgery, all surgical instruments and implant assemblies were autoclave sterilized at 250 °C for 1 hour. During surgery, the animals were gas anesthetized under 2% isoflurane in 1 L/min oxygen. For each of the nerves, a section approximately 3–4 mm was completely separated from and devoid of all connective tissue. A custom made hook (Fig. 1c, Supplementary Fig. 3a) was attached to a three-axis micromanipulator and positioned to provide a small amount of tension and suspend the nerve in the air. The slip knot was then removed, thereby releasing the silicone tube junction from the microneedle. The CNT yarn electrode assembly was then advanced into the nerve until all of the uninsulated CNT yarn segment was within the perineurium. Micro tweezers were then used to apply a small amount of pressure at the implant site while the Tungsten mirconeedle was carefully removed leaving the CNT yarn inside the nerve (Supplementary Fig. 3b). The hook was then advanced along the nerve to the next implant site, approximately 2 mm away, and the implant process was repeated. The CNT yarns and the silicone tube junctions were then carefully positioned within the incision site and completely encapsulated with 1 mL of fibrin glue (Tisseel, Baxter International Inc.) (Supplementary Fig. 3c) to secure the implant in place while the normal physiological process of collagen encapsulation took place while the fibrin glue was absorbed by the body.

The free DFT® wire ends are then tunneled and externalized at a location on the back between the shoulder blades. The free DFT® wire ends and the ground wire are then passed through a custom made, percutaneous silicone plug and soldered to an electrical connector (MCP-05-SS, Omnetics Connector Corporation). The connector was then fitted inside the silicone tube of the percutaneous plug and the tube was filled with UV curable, medical grade epoxy (OG603, Epoxy Technology Inc.).

Blunt dissection is then performed to create a pocket in the back between the skin and muscle. The implanted wires and ground wire are placed within this pocket. The percutaneous plug’s mesh base was then sutured to the underlying muscle fascia using biodegradable 3-0 sutures (Vicryl Plus, Ethicon). Finally, all skin incision sites were closed using 4-0 sutures (Webpro™, Patterson Veterinary).

### Chronic autonomic nerve recordings

The recording system was designed to accommodate two, differential recording channels. Each recording channel has eight amplifier hardware averaging for increased signal-to-noise recording results^53^. This was achieved by creating, two custom, printed circuit boards (PCB) (Supplementary Fig. 4a and b). The first PCB contains the mating connector (MCS-05-DD, Omnetics Connector Corporation) to the connector which resides in the implanted percutaneous plug. DFT® wires are soldered to make electrical connections between the two PCB boards for all the CNT yarn electrode implants (up to four total) and the implanted ground wire.

The second PCB routes each differential electrode pair to an electrical connector (NPD-36-AA-GS, Omnetics Connector Corporation) that connects directly to a commercially available 16-amplifier, neural instrumentation amplifier board (C3313/RHD2216, Intan Technologies LLC). The electrode connections are routed such that one differential channel is simultaneously recorded with amplifiers 0–7 and the other is captured with amplifiers 8–15. The RHD2216 performs the signal filtering, analog signal multiplexing, and analog to digital conversion and is controlled via a digital SPI connection to a commercially available acquisition system (C3100/RHD20000 Evaluation System, Intan Technologies, LLC) (Fig. 1e and Supplementary Fig. 4c). Neural signal acquisition was performed and stored to a laptop computer using the following system configurations: 20 k samples/sec/amplifier, DSP offset removal was enabled with the high-pass filter cutoff frequency set at 100 Hz, the amplifier bandpass filters were configured to be between 100 Hz and 9 kHz.

A manual trigger switch signal is recorded via a separate analog-to-digital input line on the RHD2000 evaluation system. This trigger signal is synchronized with the recorded neural amplifier data and is also sampled at a rate of 20 k samples/sec. Holding down a simple push button switch, produces a high logic-level trigger signal which is used to mark different events during the various experiments. Releasing the push button returns the trigger signal to a low logic-level (Supplementary Fig. 4c).

### Impedance magnitude and signal-to-noise ratio measurements and analysis

Impedance magnitude measurements were taken, over a 10 week period, between each implanted CNT yarn and an implanted ground wire at 1 kHz using a handheld meter (Protek, Z580 LCR Meter) while the animal was anaesthetized. SNR values were calculated for each recording data set over a ten week period. For each recording session, the baseline noise standard deviation was calculated using the MATLAB function *std*() between two time points where no neural activity was present. Because of the multi-unit recordings obtained with the CNT yarns, neural spikes were placed into three groups (High, Medium and Low) based on their peak-to-peak amplitudes. For each group, in each recording session, three spikes were used to calculate the voltage peak-to-peak values and divide them by the baseline noise value to determine the SNR. Minitab v17.3.1 was used to perform a linear regression analysis to determine if there was a significant change in impedance and SNR over the 10 week period using a two-tailed, paired t-test on the slope of the regression line.

### Post signal processing of acquired neural recordings

Using Matlab (version R2016b, Mathworks), the eight parallel amplifier channels of differential recording data from each CNT yarn implant pair was first averaged. The data was then processed using a 7^th^ order, zero-phase, digital bandpass filter with the lower and upper cutoff frequencies equal to 300 Hz and 1,000 Hz, respectively. Filtered data is then imported into a MATLAB algorithm that uses fuzzy c-means clustering (*fcm*) to threshold, cluster, and count spikes^54,55^. This algorithm first identifies spikes as local maxima that exceed a user-specified threshold. These spikes are then aligned with the maxima centered and 1.5 ms (30 samples) of data before and after the maximum saved for analysis. These spike waveforms are run through MATLAB’s principal component analysis function (*pca*) to reduce the dimensionality of the spikes from 61 to 5 without any significant loss in information (greater than 99% of variance is maintained using the top 5 principal components). The principal component scores associated with each spike are then processed via the MATLAB *fcm* function and sorted into clusters. The number of clusters is user-specified and is generally determined based on qualitative assessment of clusters and quantitative measures of cluster separation (i.e. L-Ratio)^56^. Firing rates for each cluster are calculated using a 2 second sliding window and are linearly interpolated using a version of MATLAB’s *envelope* function. Average firing rates were calculated during 5-second intervals before hypoxia, at the end of the hypoxic event, and after a 90-second recovery period. A two-tailed, paired t-test was then conducted to compare firing rates of each spike cluster at each of these three time-points.

Spike sorting analysis was confirmed using the spike sorting package UltraMegaSort2000 (UMS2000), described previously by Hill *et al*.^41^. Spikes were separated using the splitmerge_tool GUI, and plots showing the spikes in feature space, spike time correlations, and cluster firing rates were generated using the UMS2000 plot functions (plot_features, plot_xcorr, plot_isi, plot_stability). Cluster quality metrics (fractions of false negative and false positive events) were calculated for each day using the UMS2000 functions, and are displayed in Tables 1 and 2. Refractory period violations were calculated with ss_rpv_contamination; undetected spike fractions were calculated using ss_undetected; censored spike fractions were calculated using ss_censored; and overlap of clusters was calculated using ss_gaussian_overlap.

### Histology

Nerves were extracted from rats after implantation, and were immediately placed into hydrogel monomer solution (clarityresourcecenter.org). After incubation in the hydrogel monomer solution for 2–3 weeks at 4 °C, samples were then polymerized at 37 °C and low oxygen (a layer of peanut oil was added to the tube to decrease the concentration of oxygen in the solution). After polymerization, the excess gel was removed, and the nerve is placed into clearing solution for passive clearing, as described on the Clarity Resource Forum^57^. Primary antibodies (W and X) were diluted 1:500 in 1 mL of PBST. After a 1-day wash in PBST, the sample was incubated in secondary antibody solution, which contained secondary antibodies (Y and Z), diluted 1:500, and DAPI, diluted 1:1000. After another 1-day PBST wash PBST, the sample was placed in VectaShield with DAPI (Vector Laboratories) on a glass-bottom petri dish (Ted Pella, Inc.). Samples were imaged on a Leica SP8 gSTED Super-Resolution Confocal microscope (Leica Microsystems).

### Chronic sciatic nerve charge threshold stimulation

Five Sprague Dawley rats each had two CNT yarn electrodes implanted into their tibial and peroneal fascicles, and stimulation thresholds were subsequently measured. While anesthetized under isoflourane, biphasic square pulses were applied differentially using an isolated stimulator (A-M Systems Analog Isolator 2200) to these implanted electrodes (i.e. cathode and anode both in the fascicle) while the leg was monitored via visual inspection and measured electromyogram (EMG) response. One hertz stimulation was performed to avoid muscle fatigue. Each phase of the stimulation lasted 100 µs with no inter-phase interval.

### Data Availability

The datasets generated during and/or analysed during the current study are available from the corresponding author on reasonable request.

## Electronic supplementary material

Supplementary Information
