# Supplementary material for: Chronic interfacing with the autonomic nervous system using carbon nanotube (CNT) yarn electrodes
Source: Sci Rep. 2017 Sep 15;7:11723. doi: 10.1038/s41598-017-10639-w (PMC5601469; doi:10.1038/s41598-017-10639-w)
Supplement: Supplementary file 1 — Supplementary Information [file 41598_2017_10639_MOESM1_ESM.pdf]

Chronic interfacing with the autonomic nervous system using carbon nanotube (CNT) yarn electrodes

Grant A. McCallum[1], Xiaohong Sui[3], Chen Qiu[1], Joseph Marmerstein[1],Yang Zheng[1], Thomas E. Eggers[1], Chuangang Hu[2], Liming Dai[2], Dominique M. Durand[1,*]

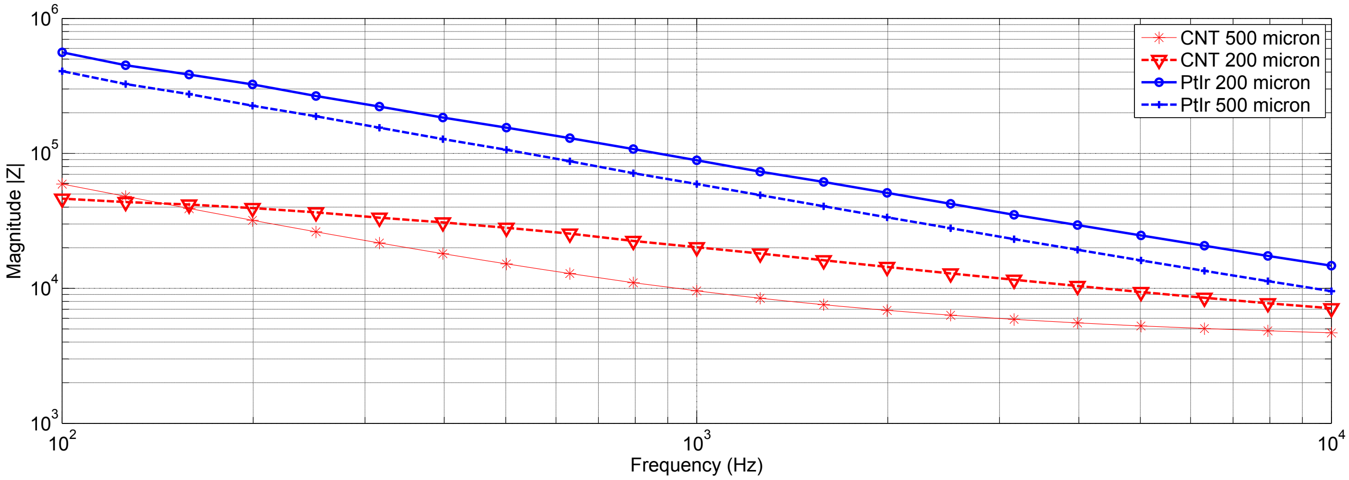

a

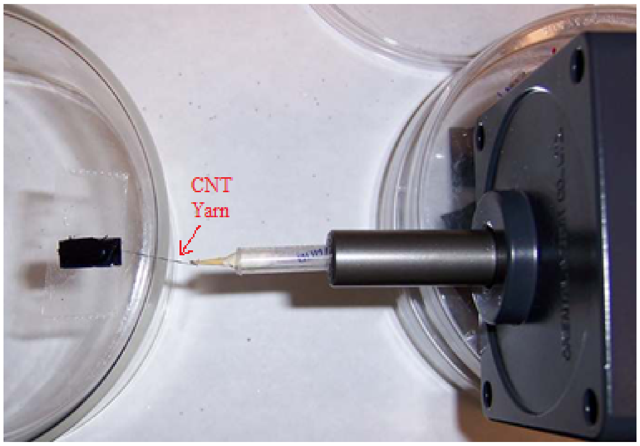

b

Supplementary Figure 1 | CNT yarn vs PtIr wire electrical and mechanical comparison and CNT yarn fabrication **a**, Electrochemical impedance spectroscopy measurements comparing CNT yarns and PtIr wires of different exposed lengths (200 and 500 micron) with the same 10 micron diameters. **b**, Photograph showing the fabrication process by spinning VA-MWCNT arrays to form CNT yarns.

[1]Neural Engineering Center, Department of Biomedical Engineering, Case Western Reserve University, Cleveland, OH 44106-7078 USA, [2]Department of Macromolecular Science and Engineering, Case Western Reserve University, Cleveland, OH 44106-7078 USA, [3]School of Biomedical Engineering, Shanghai Jiao Tong University, Shanghai 200240, China. *Correspondence should be addressed to D. Durand (dxd6@case.edu)

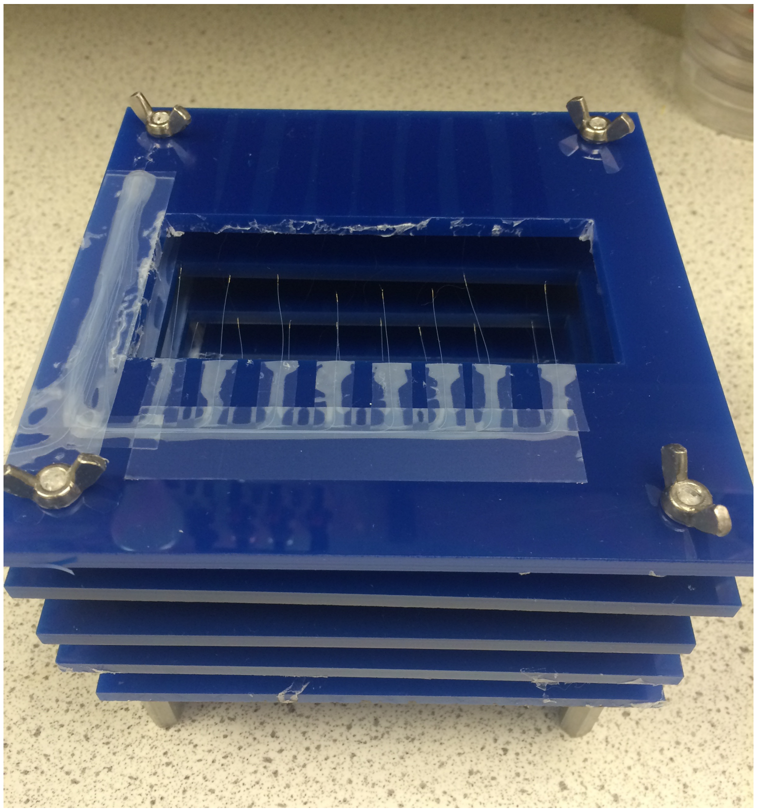

a

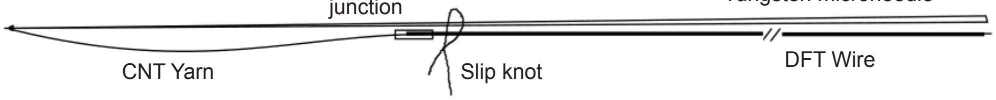

b

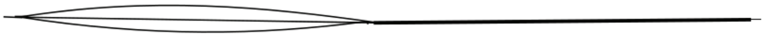

c

Supplementary Figure 2 | Wire rack for Parylene-C coating and diagrams of electrode assembly. a, Multi-layer rack for holding CNT yarns for insulation vapor deposition. The CNT yarn/DFT wire junction is placed in the middle of the window and tape is used to mask the CNT yarn segment that will be the exposed electrode site. **b**, Diagram showing fully assembled electrode and needle inserter. **c**, Diagram showing the assembled ground wire electrode.

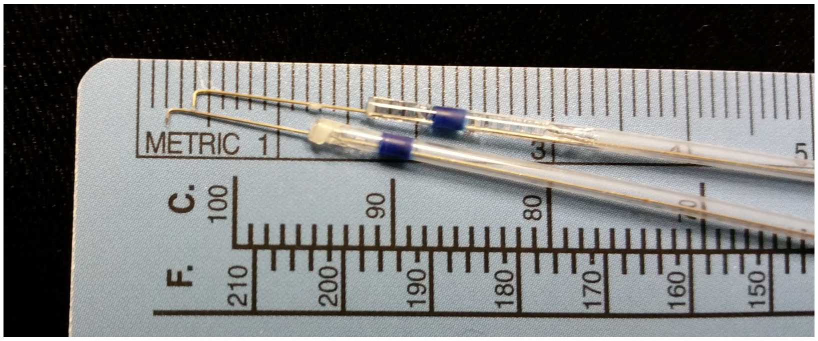

a

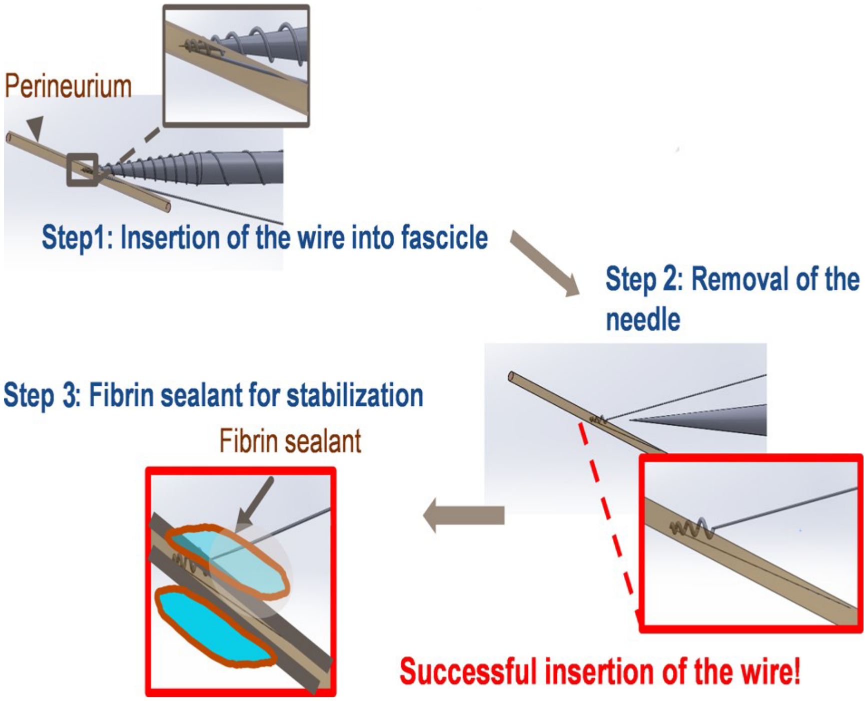

b

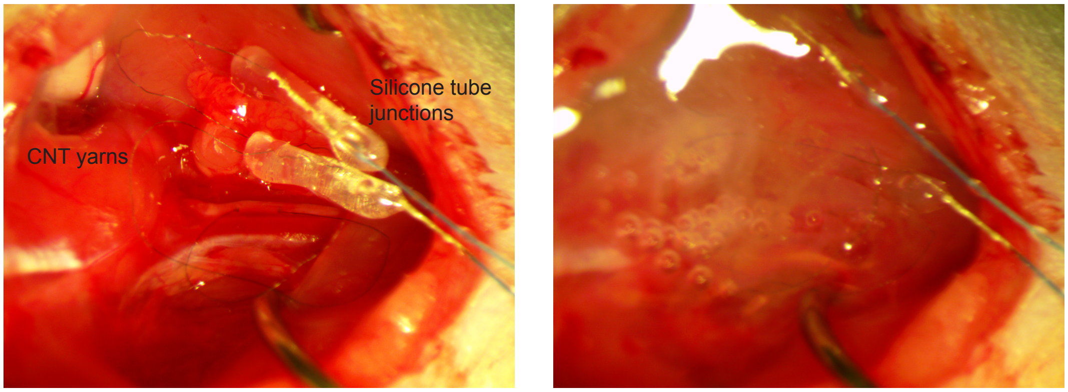

c

Supplementary Figure 3 | CNT yarn peripheral nerve implant process. **a**, Custom made Teflon coated, Tungsten hooks used to hold the nerve during the insertion process. **b**, Diagram showing the CNT yarn insertion process in a peripheral nerve. **c**, Images showing the before and after applying Tisseel (fibrin glue) to secure the CNT yarn implants and silicone junctions.

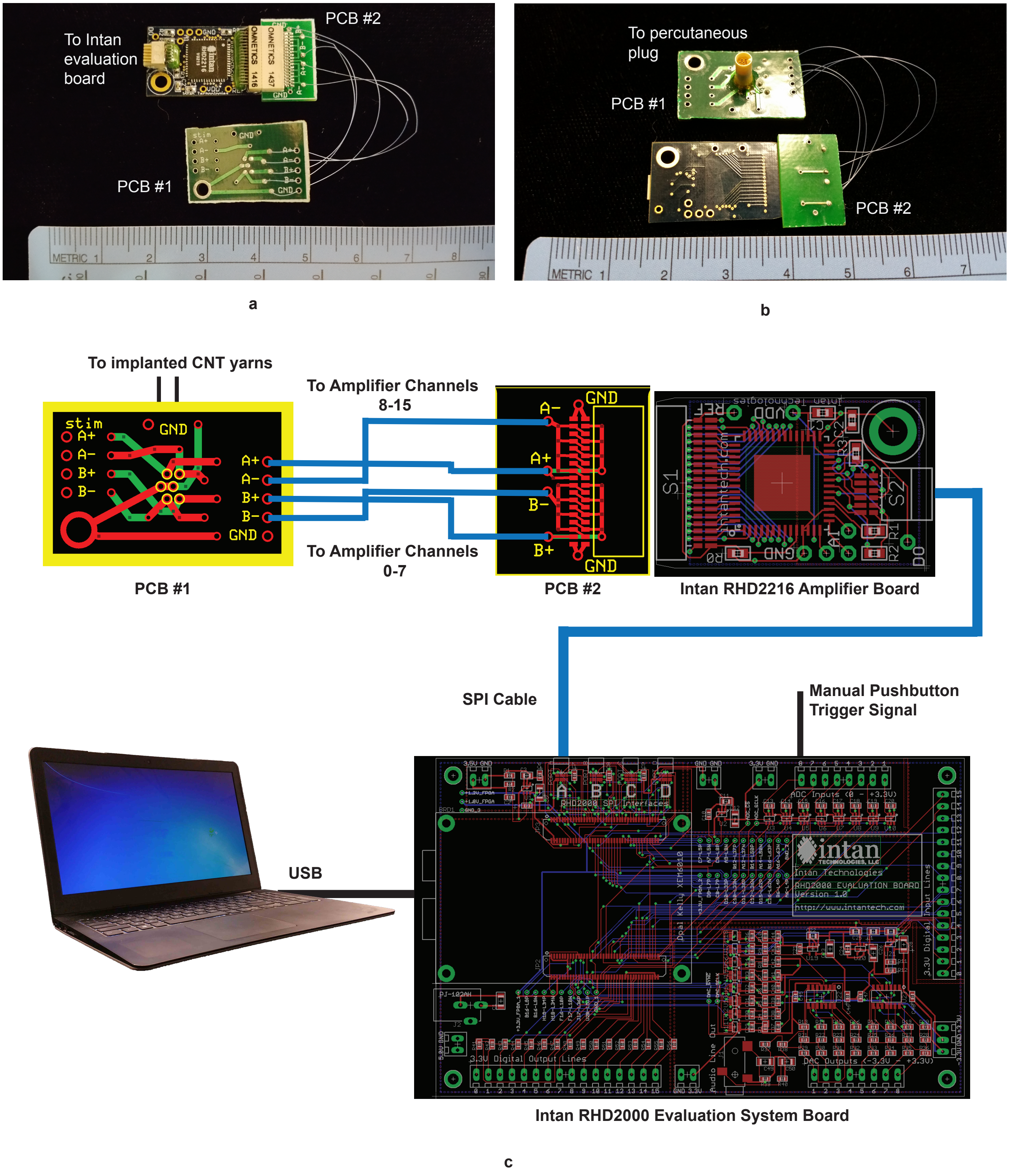

Supplementary Figure 4 | Electronics system for recording peripheral nerve activity **a**, Top-side of external PCB boards used for neural signal recording. PCB #1 connects directly to the implanted percutaneous plug on the rat. PCB #2 connects directly to the Intan amplifier board. **b**, Bottom-side of exteral PCB boards. **c**, Diagram showing the entire recording path from the CNT yarn implants through PCB #1 to PCB #2 that routes the signals to multiple Intan amplifier inputs for hardware averaging. A SPI cable connects the external PCB boards to the Intan evaluation system for processing and storage to a laptop via a USB connection. Also shown is the manual pushbutton connection to an ADC input on the Intan evaluation system to enable trigger signal recording during the experiments.

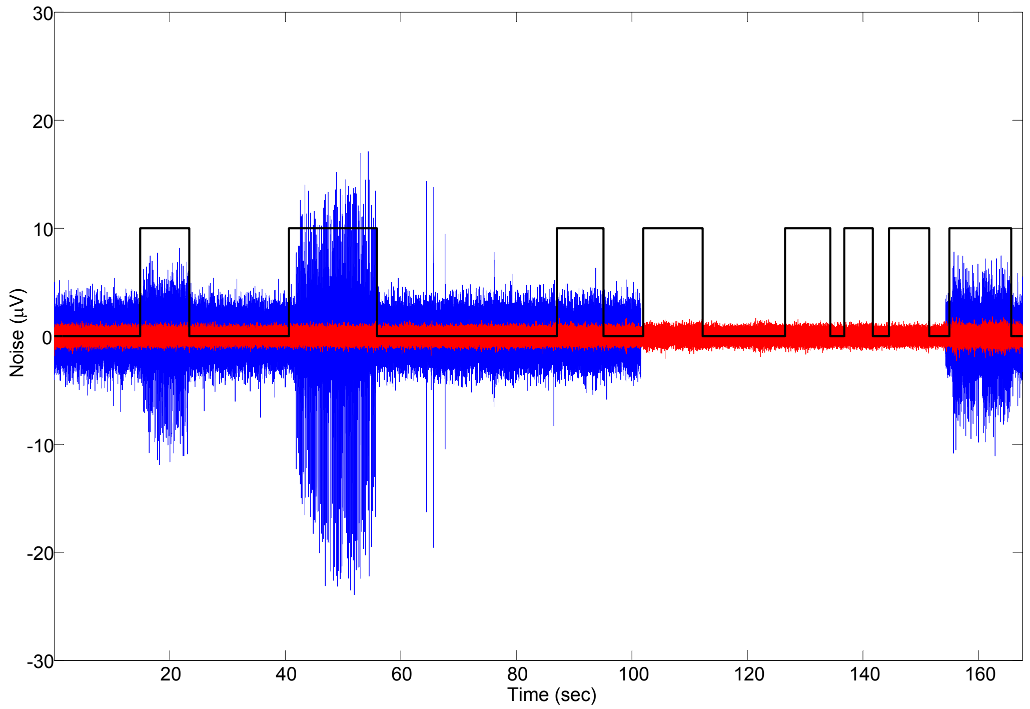

a

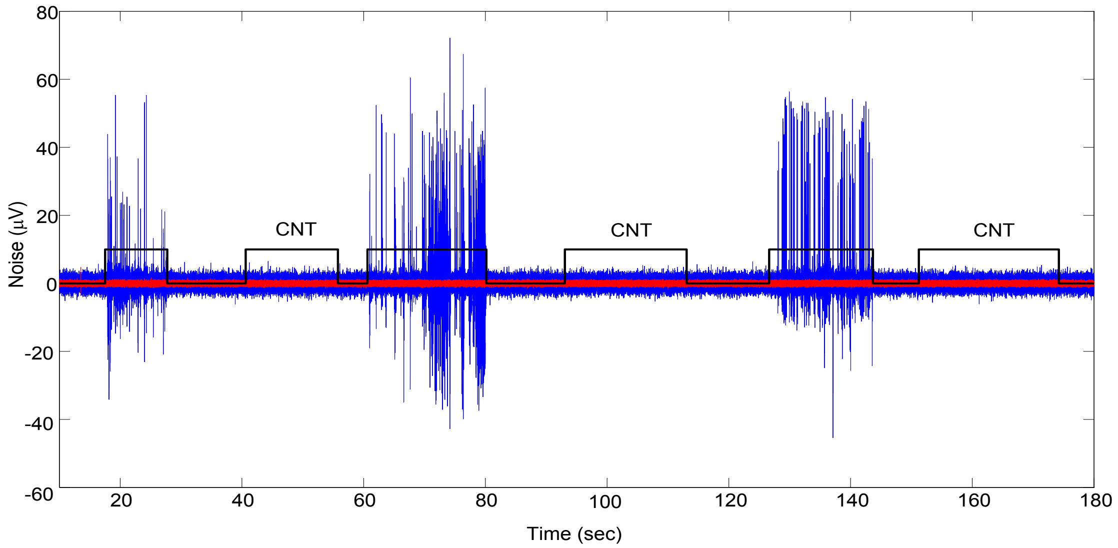

b

Supplementary Figure 5 | Triboelectric noise contamination in commercial implant electrodes vs CNT yarns. Both a commecially available sympathetic nerve activity (SNA) recording implant system (Millar Inc. - TRM56P) and two CNT yarn electrodes were placed in a glass saline tank. The CNT yarn electrodes were connected externally to differential Intan amplifiers. Both recorded signals were synchonized together by using a trigger from the Intan system to the PowerLab/Millar system. Blue traces represent the Millar electrode recordings and red traces are recordings from the CNT yarn electrodes. The black trace indicates when vibration/movement was applied to the electrode leads. **a**, A vibration motor source (Sparkfun - ROB-08449) was applied to the external saline tank wall in various locations to induce electrode movement. The CNT yarn baseline remains constant with no spike artifact while the Millar electrodes produce large spike artifacts from the motion. **b**, Manual motion of the leads was done using a wooden stick by alternating between the Millar electrodes and the CNT yarns starting with the Millar system as shown. Once again, the CNT yarn signals have a constant noise level with no spike artifacts compared to the Millar electrodes.

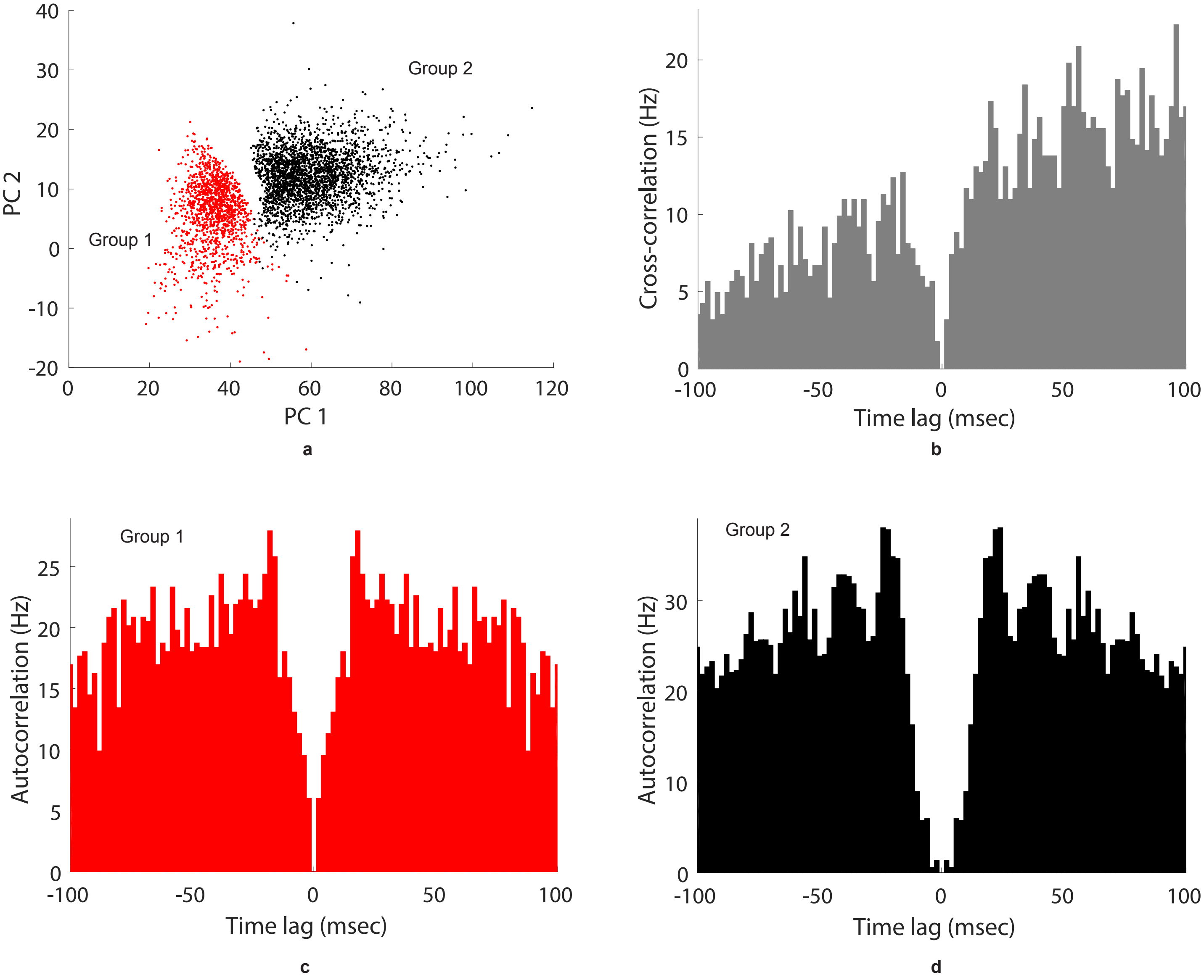

Supplementary Figure 6 | Spike cluster separation 7 days post-implant. Several spike separation and quality metrics calculated from data collected 7 days after electrode implantation. **a,** Representation of clusters 1 and 2 in the feature space, showing only the first two principal components for each spike. **b,** Cross-correlation of spike times between clusters 1 and 2; bin width is 2ms. **c,** Auto-correlation of spike times from cluster 1; bin width is 2 ms. **d,** Auto-correlation of spike times from cluster 2; bin width is 2 ms.

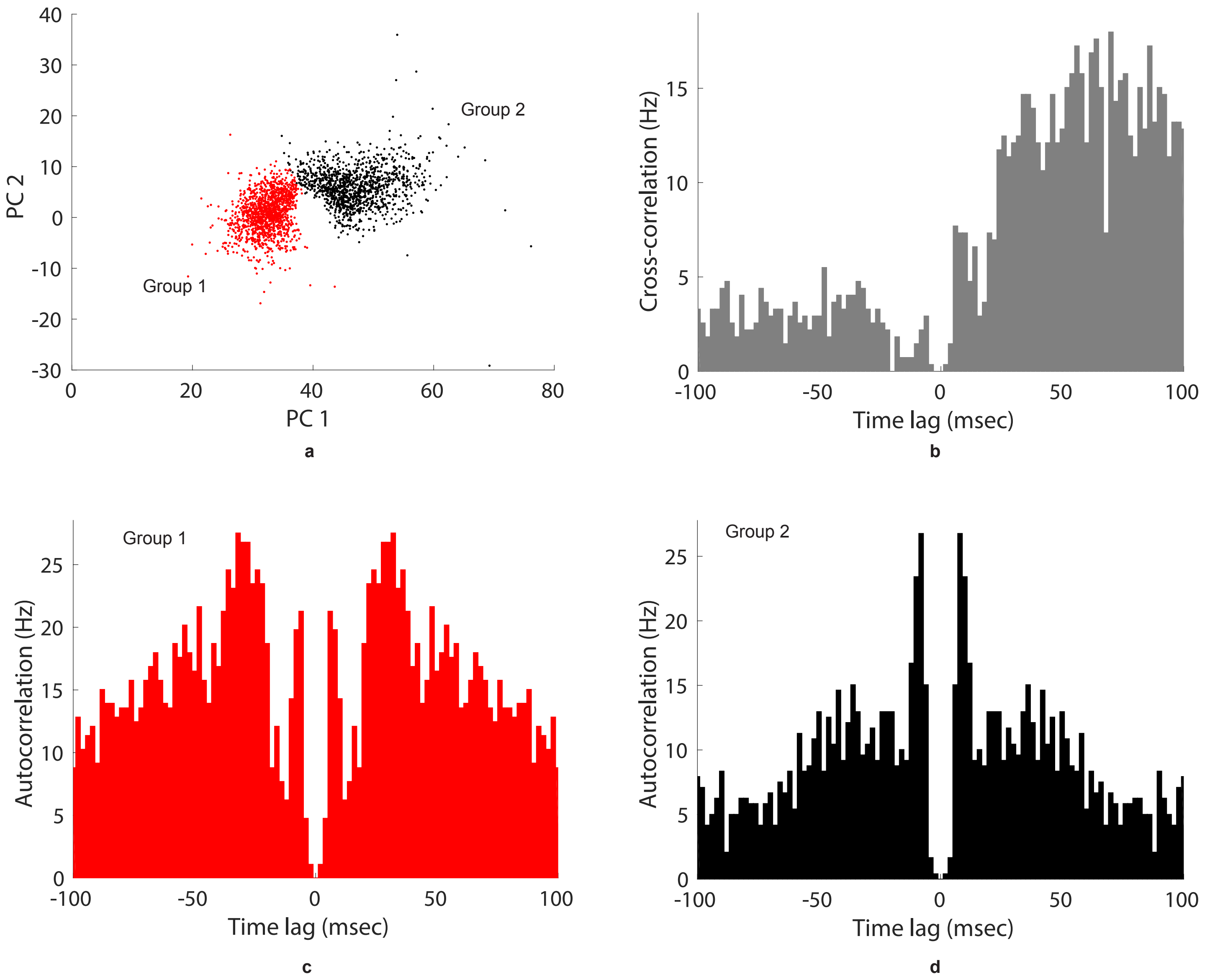

Supplementary Figure 7 | Spike cluster separation 16 days post-implant. Several spike separation and quality metrics calculated from data collected 16 days after electrode implantation. **a,** Representation of clusters 1 and 2 in the feature space, showing only the first two principal components for each spike. **b,** Cross-correlation of spike times between clusters 1 and 2; bin width is 2ms. **c,** Auto-correlation of spike times from cluster 1; bin width is 2 ms. **d,** Auto-correlation of spike times from cluster 2; bin width is 2 ms.
